# Intraperitoneal dedifferentiated liposarcoma showing MDM2 amplification: case report

**DOI:** 10.1186/1477-7819-11-305

**Published:** 2013-11-26

**Authors:** Carlo Grifasi, Armando Calogero, Nicola Carlomagno, Severo Campione, Francesco Paolo D’Armiento, Andrea Renda

**Affiliations:** 1 Department of Advanced Biomedical Sciences, Section of General Surgery, University of Naples Federico II, Naples, Italy; 2 Department of Advanced Biomedical Sciences, Section of Anatomic Pathology, University of Naples Federico II, Naples, Italy; 3 Department of Advanced Biomedical Sciences, University of Naples Federico II, Via Pansini 5, 80131 Naples, Italy

**Keywords:** Dedifferentiated liposarcoma, Intraperitoneal location, Surgery, Histopathology, *MDM2*

## Abstract

**Background:**

Liposarcoma is the most common type of soft tissue sarcoma (STS). It is divided into five groups according to histological pattern: well-differentiated, myxoid, round cell, pleomorphic, and dedifferentiated. Dedifferentiated liposarcoma most commonly occurs in the retroperitoneum, while an intraperitoneal location is extremely rare. Only seven cases have been reported in literature. Many pathologists recognize that a large number of intra-abdominal poorly differentiated sarcomas are dedifferentiated liposarcomas. We report a case initially diagnosed as undifferentiated sarcoma that was reclassified as intraperitoneal dedifferentiated liposarcoma showing an amplification of the *MDM2* gene.

**Case presentation:**

A 59-year-old woman with abdominal pain and constipation was referred to the Department of Advanced Biomedical Sciences, University of Naples Federico II, Naples, Italy, in November 2012. On physical examination, a very large firm mass was palpable in the meso-hypogastrium. Computed tomography (CT) scan showed a heterogeneous density mass (measuring 10 × 19 cm) that was contiguous with the mesentery and compressed the third part of the duodenum and jejunum.

At laparotomy, a large mass occupying the entire abdomen was found, adhering to the first jejunal loop and involving the mesentery. Surgical removal of the tumor along with a jejunal resection was performed because the first jejunal loop was firmly attached to the tumor.

Macroscopic examination showed a solid, whitish, cerebroid, and myxoid mass, with variable hemorrhage and cystic degeneration, measuring 26 × 19 × 5 cm. Microscopic examination revealed two main different morphologic patterns: areas with spindle cells in a myxoid matrix and areas with pleomorphic cells. The case was initially diagnosed as undifferentiated pleomorphic sarcoma. Histological review showed areas of well-differentiated liposarcoma. Fluorescence *in situ* hybridization (FISH) analysis was performed and demonstrated an amplification of the *MDM2* gene. Definitive diagnosis was intraperitoneal dedifferentiated liposarcoma.

No adjuvant therapy was given, but 5 months after laparotomy, the patient presented with a locoregional recurrence and chemotherapy with high-dose ifosfamide was started.

**Conclusions:**

No guidelines are available for the management of intraperitoneal dedifferentiated liposarcoma. We report this case to permit the collection of a larger number of cases to improve understanding and management of this tumor. Moreover, this study strongly suggests that poorly differentiated sarcomas should prompt extensive sampling to demonstrate a well-differentiated liposarcoma component and, if possible, FISH analysis.

## Background

Soft tissue sarcoma (STS) is a rare mesenchymal tumor arising from non-epithelial connective tissue sources. The incidence of STS is approximately 4 to 5/100,000/year in Europe [1-3]. Liposarcoma is the most common STS, and can bedivided into five groups according to histological pattern: well-differentiated, myxoid, round cell, pleomorphic, and dedifferentiated [4-7].

Dedifferentiated liposarcoma is a variant of liposarcoma with a worse prognosis and it most commonly occurs in the retroperitoneum, while an intraperitoneal location is extremely rare. Five cases of dedifferentiated liposarcoma in small bowel mesentery have been described [8]. Moreover, a case of dedifferentiated liposarcoma has been documented in the sigmoid mesocolon [9]. Another case reported in the literature was located at an intraperitoneal location [10]. Many pathologists recognize that a large number of intra-abdominal poorly differentiated sarcomas are dedifferentiated liposarcomas [11].

We report herein a case initially diagnosed as undifferentiated sarcoma that was reclassified as intraperitoneal dedifferentiated liposarcoma showing an amplification of the murine double minute 2 (*MDM2*) gene, in order to emphasize that in most cases of undifferentiated sarcomas, a specific line of differentiation can be demonstrated.

## Case presentation

A 59-year-old woman with Parkinson’s disease, who complained of abdominal pain and constipation, was referred to the Department of Advanced Biomedical Sciences, University of Naples Federico II, Naples, Italy, in November 2012. On physical examination, a firm mass was palpable in the meso-hypogastrium. There were no laboratory abnormalities, except hyposideremia (29 mcg/dL) and a positive test result for HBsAg. Tumor markers such as carcinoembryonic antigen (CEA), CA19-9, and CA-125 were within their normal ranges.

**Figure 1:**
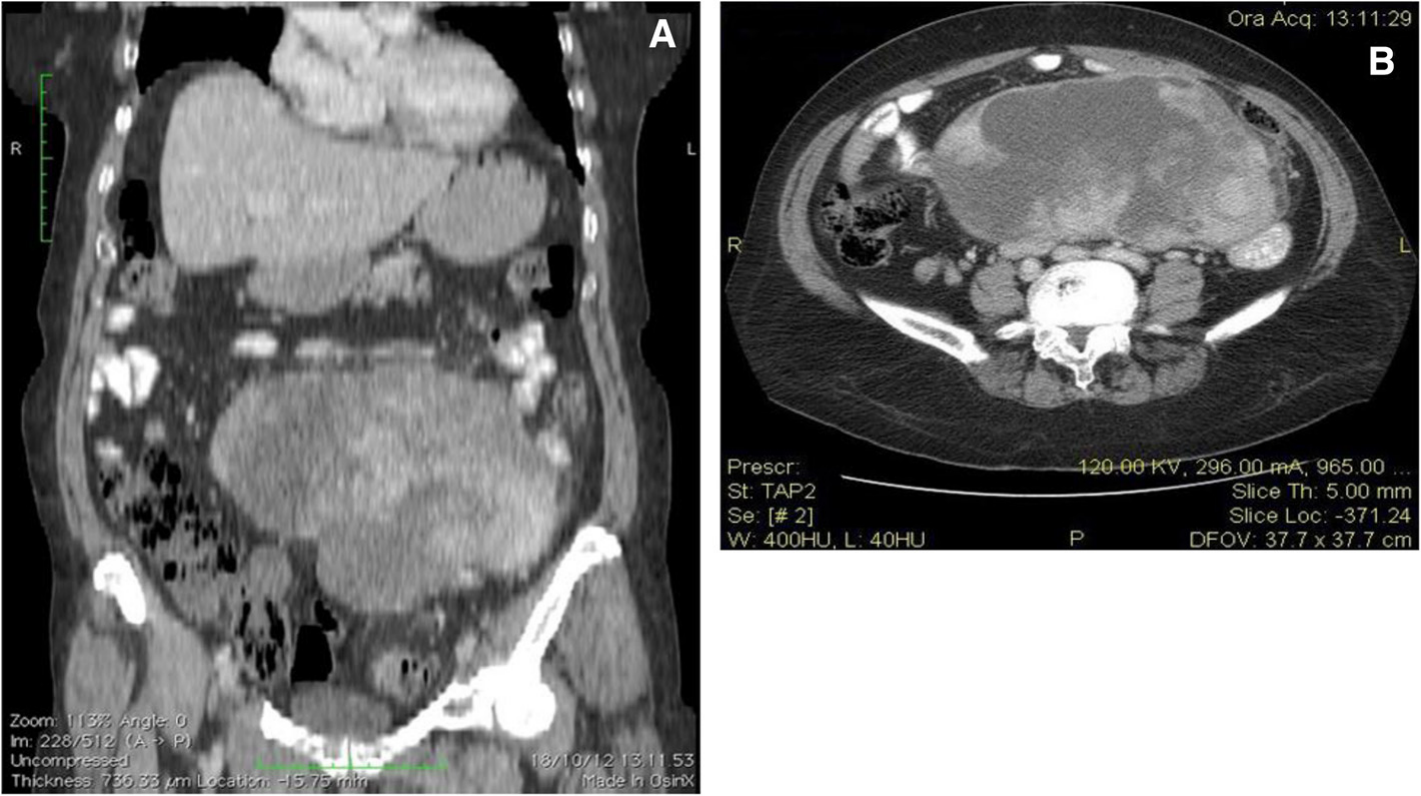
**CT scans. (A)** Coronal CT image demonstrating the extent of the mass; and **(B)** CT slice showing the heterogeneous density of the mass with lateral displacement of the bowel.

Ultrasound revealed a very large mass with a complex echogenicity, almost completely occupying the lower quadrants of the abdomen. Computed tomography (CT) scan showed that the mass occupied the entire meso-hypogastric region, had heterogeneous density, involved the mesentery, and displaced the small bowel loops (Figure 1). Moreover, it also showed a complicated diverticulitis at the sigmoid colon with a pericolic abscess.

**Figure 2:**
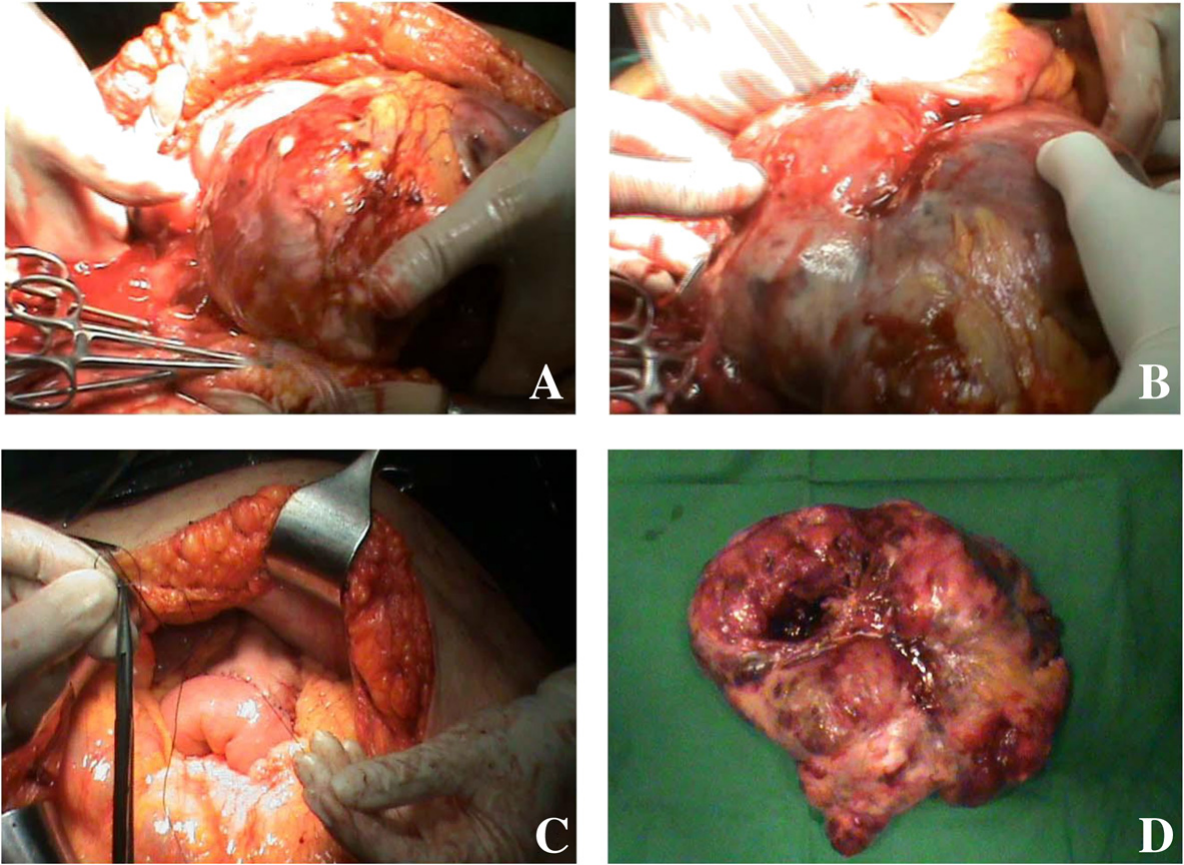
**Images of the tumor. (A)** The very large tumor; **(B)** tumor adhering to the small bowel loops; **(C)** gastrojejunostomy; and **(D)** the surgical specimen.

At laparotomy a very large mass was found with dense adhesions to the mesentery and sigmoid colon. The tumor seemed to arise from the first jejunal loop. A jejunal resection with mass excision and a gastrojejunostomy was performed (Figure 2). We also made an anterior resection with primary anastomosis for the sigmoid complicated diverticulitis, together with cholecystectomy for cholelithiasis and appendicectomy.

**Figure 3:**
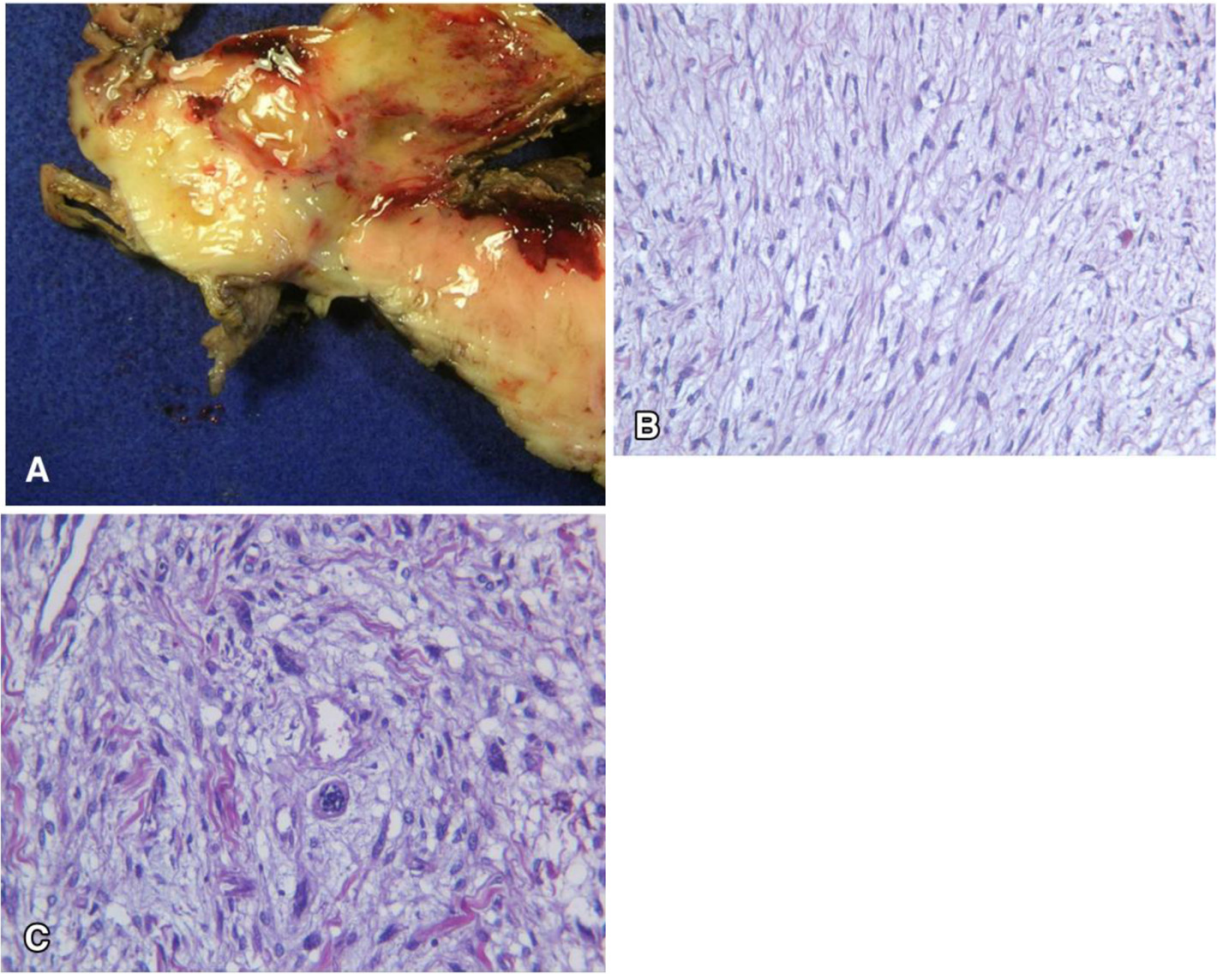
**Macroscopic and microscopic examination. (A)** Heterogeneous cut surface; **(B)** a population of spindle cells in a myxoid matrix; and **(C)** a population of pleomorphic cells.

Macroscopic examination of the specimen showed a very large, solid, whitish, cerebroid, and myxoid mass, with variable hemorrhage and cystic degeneration, a heterogeneous cut surface, and measuring approximately 26 × 19 × 5 cm. At an extremity there was a small intestinal resection of 10 cm that was on the serous surface of the tumor (Figure 3A).

Microscopic examination revealed two main morphologic patterns: first, areas that were less crowded with spindle cells in a myxoid matrix (Figure 3B) and occasionally disposed in a storiform pattern; and second, more crowded areas with pleomorphic cells and atypical mitosis (Figure 3C). Histological examination also revealed that the neoplasia reached the muscularis propria region of the intestine.

Immunohistochemical stains for cytokeratin, CD34, S100, actin, calretinin, and CD117 (c-Kit) were negative, while Bcl-2, EMA, and CD99 were positive in the neoplastic cells.The histopathological diagnosis was of an undifferentiated pleomorphic sarcoma.

Histological review showed areas of well-differentiated liposarcoma and the tumor was reclassified as dedifferentiated liposarcoma. Moreover, a fluorescence *in situ* hybridization (FISH)analysis was performed and demonstrated an amplification of the *MDM2* gene. These findings were consistent with an intraperitoneal dedifferentiated liposarcoma.

## Discussion

Intraperitoneal dedifferentiated liposarcomas are rare and, to our knowledge, only eight cases have been reported to date (Table 1). Of these cases, patients were aged between 59 and 63 years, and five were female. There were no characteristic complaints at presentation and patients reported abdominal fullness or pain. The diagnosis of intraperitoneal dedifferentiated liposarcoma is always late as the disease remains asymptomatic until progression reaches the end stages.

**Table 1:** Reported cases of intraperitoneal dedifferentiated liposarcoma

**Reference**	**Age (years)/gender**	**Site**	**Size (cm)**	**Primary treatment**	**Adjuvant therapy**	**Period to first recurrence (months)**	**Outcome**
Hasegawa [8]	59/M	Mesentery	14	Surgery	No	72	DOD, 6 years, 1 month
Hasegawa [8]	58/F	Mesentery	20	Surgery	No	50	NED, 9 years, 7 month
Hasegawa [8]	56/F	Mesentery	30	Surgery	No	12	DOD, 6 years, 2 months
Hasegawa [8]	52/F	Mesentery	40	Surgery	No	159	DOD, 20 years, 3 months
Hasegawa [8]	63/F	Mesentery	10	Surgery	No	124	AWD, 15 years, 2 month
Karaman [10]	62/M	Intraperitoneal location	10	Surgery	RTX	0	AWD, 1 year, 3 months
Winn [9]	59/M	Sigmoid mesocolon	25	Surgery	No	30	NED, 4 years, 6 months
Current case report	59/F	Intraperitoneal location	26	Surgery	No	5	AWD, 7 months

AWD, alive with disease; DOD, died of disease; NED, no evidence of disease; RTX, radiation therapy.

Imaging modalities may help in preoperative diagnosis of the origin of tumor. However, CT findings may suggest the diagnosis of liposarcoma when the tumor contains areas of fat attenuation, which is less likely to be seen in higher-grade liposarcomas. Moreover, dedifferentiated liposarcomas demonstrate marked heterogeneity on magnetic resonance imaging (MRI), with areas of necrosis and heterogeneous contrast enhancement, indistinguishable from other high-grade sarcomas [12-15].

Accurate diagnosis demands an experienced pathologist, the use of immunohistochemistry, and often also cytogenetics [16,17]. Microscopically, this case resembled an undifferentiated pleomorphic sarcoma, which could not be a specific soft tissue tumor but a common morphologic appearance resulting from tumor progression of various sarcomas, especially liposarcomas, but also others. Dedifferentiated liposarcomas typically show extensive areas resembling undifferentiated pleomorphic sarcoma; the diagnosis of dedifferentiated liposarcoma is thus reached by identification of areas of well-differentiated liposarcoma, but this component can be scarce and therefore can be lost by sampling or simply not present [18]. Consequently, dedifferentiated liposarcomas may exist without any demonstrable well-differentiated liposarcoma component.

Well-differentiated liposarcomas/atypical lipomatous tumors and dedifferentiated liposarcomas have been cytogenetically shown to harbor ring and giant marker chromosomes consisting of amplicons in the 12q13-15 region, resulting in amplification of several genes, including most notably *MDM2*[19,20]. Identifying *MDM2* amplification by immunohistochemistry, FISH, quantitative PCR, or comparative genomic hybridization (CGH) may prove an adjunctive tool in the diagnosis of lipomatous neoplasms. This feature can be of remarkable help in reaching the diagnosis of dedifferentiated liposarcoma, particularly in cases of poorly differentiated sarcomas for which a specific line of differentiation cannot be demonstrated, as observed in this case.

Radical excision of the tumor offers the possibility of longer survival and a disease-free interval. In all patients reviewed in the literature, surgical excision was the first line of treatment of intraperitoneal liposarcoma. The value of adjuvant chemotherapy has not been established. Postoperative radiotherapy to the whole tumor bed is not feasible at a tolerable toxicity [21,22]. One patient underwent adjuvant radiotherapy and was asymptomatic for the 15-month follow-up period (Table 1).

In terms of recurrence, follow-up information is available for all cases reported. Only one patient did not have recurrence. Of the eight patients, three died of disease.

## Conclusions

In summary, the rarity of intraperitoneal dedifferentiated liposarcoma makes it difficult to investigate clinical characteristics, therapy, and outcomes. Differential diagnosis of this tumor can be very difficult because the histological examination does not demonstrate a specific line of differentiation. Therefore, in cases initially diagnosed as poorly differentiated sarcomas, extensive sampling and an analysis of *MDM2* amplification using FISH are recommended.Surgery is the main treatment and the role of adjuvant therapy is widely debated.In order to improve overall understanding of the intraperitoneal liposarcoma, it is useful to analyze the collected data of all observed cases.

## Consent

Written informed consent was obtained from the patient for publication of this case report and any accompanying images. A copy of the written consent is available for review by the Editor-in-Chief of this journal.

## Abbreviations

CEA: Carcinoembryonic antigen; CGH: Comparative genomic hybridization; CT: Computed tomography; FISH: Fluorescence *in situ* hybridization; MDM2: Murine double minute 2; MRI: Magnetic resonance imaging; PCR: Polymerase chain reaction; STS: Soft tissue sarcoma.

## Competing interests

The authors declare that they have no competing interests.

## Authors’ contributions

CG carried out acquisition, analysis and interpretation of data, and drafted the manuscript. AC and NC critically revised the study. SC made substantial contributions to acquisition, analysis, and interpretation of histopathological data. FPD carried out histopathological examination and critically revised the study. AR performed the surgical operation, conceived the study, and participated in its design and coordination. All authors read and approved the final manuscript.

## Authors’ information

CG: specializzando in Chirurgia Generale, Dipartimento di Scienze Biomediche Avanzate, Università Federico II, Naples, Italy; AC: specialista in Chirurgia dell’ apparato digerente ed endoscopia digestiva chirurgica, Dipartimento di Scienze Biomediche Avanzate, Università Federico II, Naples, Italy; NC: dirigente medico, Dipartimento di Scienze Biomediche Avanzate, Università Federico II, Naples, Italy; SC: specializzando in Anatomia Patologica, Dipartimento di Scienze Biomediche Avanzate, Università Federico II, Naples, Italy; FPD: professore ordinario di Anatomia Patologica, Dipartimento di Scienze Biomediche Avanzate, Università Federico II, Naples, Italy; and AR:professore ordinario di Chirurgia Generale, Dipartimento di Scienze Biomediche Avanzate, Università Federico II, Naples, Italy.
